# Topology and expressed repertoire of the *Felis catus* T cell receptor loci

**DOI:** 10.1186/s12864-019-6431-5

**Published:** 2020-01-06

**Authors:** Araya Radtanakatikanon, Stefan M. Keller, Nikos Darzentas, Peter F. Moore, Géraldine Folch, Viviane Nguefack Ngoune, Marie-Paule Lefranc, William Vernau

**Affiliations:** 1 Department of Pathology, Microbiology and Immunology, School of Veterinary Medicine, University of California, Davis, CA USA; 2 Department of Pathobiology, Ontario Veterinary College, University of Guelph, Guelph, Ontario Canada; 3 Department of Internal Medicine II, University Hospital Schleswig-Holstein, Kiel, Germany; 4 Central European Institute of Technology, Masaryk University, Brno, Czech Republic; 5 IMGT® the international ImMunoGeneTics information system®, Laboratoire d’ImmunoGénétique Moléculaire LIGM, Institut de Génétique Humaine IGH, UMR 9002 CNRS, Université de Montpellier, Montpellier Cedex 5, France

**Keywords:** Feline, T cell receptor, TRG, TRB, TRA/TRD, Expressed repertoire, V/J usage

## Abstract

**Background:**

The domestic cat (*Felis catus*) is an important companion animal and is used as a large animal model for human disease. However, the comprehensive study of adaptive immunity in this species is hampered by the lack of data on lymphocyte antigen receptor genes and usage. The objectives of this study were to annotate the feline T cell receptor (TR) loci and to characterize the expressed repertoire in lymphoid organs of normal cats using high-throughput sequencing.

**Results:**

The *Felis catus* TRG locus contains 30 genes: 12 TRGV, 12 TRGJ and 6 TRGC, the TRB locus contains 48 genes: 33 TRBV, 2 TRBD, 11 TRBJ, 2 TRBC, the TRD locus contains 19 genes: 11 TRDV, 2 TRDD, 5 TRDJ, 1 TRDC, and the TRA locus contains 127 genes: 62 TRAV, 64 TRAJ, 1 TRAC. Functional feline V genes form monophyletic clades with their orthologs, and clustering of multimember subgroups frequently occurs in V genes located at the 5′ end of TR loci. Recombination signal (RS) sequences of the heptamer and nonamer of functional V and J genes are highly conserved. Analysis of the TRG expressed repertoire showed preferential intra-cassette over inter-cassette rearrangements and dominant usage of the TRGV2–1 and TRGJ1–2 genes. The usage of TRBV genes showed minor bias but TRBJ genes of the second J-C-cluster were more commonly rearranged than TRBJ genes of the first cluster. The TRA/TRD V genes almost exclusively rearranged to J genes within their locus. The TRAV/TRAJ gene usage was relatively balanced while the TRD repertoire was dominated by TRDJ3.

**Conclusions:**

This is the first description of all TR loci in the cat. The genomic organization of feline TR loci was similar to that of previously described jawed vertebrates (*gnathostomata*) and is compatible with the birth-and-death model of evolution. The large-scale characterization of feline TR genes provides comprehensive baseline data on immune repertoires in healthy cats and will facilitate the development of improved reagents for the diagnosis of lymphoproliferative diseases in cats. In addition, these data might benefit studies using cats as a large animal model for human disease.

## Background

T cells are crucial for effective immune responses to both microbial infection and cancer, and mediate their function through highly diverse surface receptor specificities. T cells can be divided into two distinct lineages, alpha/beta (αβ) or gamma/delta (γδ). The T cell receptor (TR) protein chains are encoded by four TR loci, TR beta (TRB), TR gamma (TRG) and the intertwined TR alpha (TRA) and TR delta (TRD) loci [1]. The complete TRB and TRD chains are encoded by variable (V), diversity (D), joining (J) and constant (C) genes whereas TRA and TRG chains lack a D gene component [2]. The C domain of the TR is anchored to the cell membrane, while the V domain, encoded by rearranged germline V, D and J genes, is responsible for peptide and major histocompatibility (MH) recognition [3]. The size of the TR expressed diversity is estimated at 2 × 10^7^ in humans and 2 × 10^6^ in mice [4]. The huge potential diversity (estimates from 10^12^ to 10^18^) of the antigen receptors, immunoglobulins (IG) or antibodies and TR, is generated from a limited number of germline sequences through rearrangement of V, (D), and J genes [1, 2, 5, 6]. This process is guided through recombination signaling (RS) sequences flanking the V, D, and J genes [6]. The lymphocyte specific endonuclease recombinase activating genes (composed of RAG1 and RAG2) initiate non-homologous end-joining (NHEJ) by breaking double-stranded DNA between the coding regions and their adjacent RS. Multiple repair proteins sequentially assist with the completion of the NHEJ process. Junctional diversity is introduced by an exonuclease which removes nucleotides at the 3′ or 5′ end of the coding region of the genes which rearrange and by the template-independent DNA polymerase called terminal deoxynucleotidyl transferase (TdT) which randomly adds nucleotides not encoded in the germline genomes and creates the N-diversity regions [7]. The resulting hypervariable region is referred to as the complementarity determining region 3 (CDR3), which forms, with the germline encoded CDR1 and CDR2, the antigen-binding site and determines the specificity of the antigen receptor.

The annotation of IG and TR loci is challenging because V, D and J genes do not have the classical intron/exon structure that is detected by standard gene annotation pipelines. In addition, certain gene types such as the J gene and especially the D gene, are very short. IMGT®, the international ImMunoGeneTics information system® (IMGT) has provided the standardized scientific rules for the identification (keywords), classification (subgroup, gene and allele nomenclature), description (labels) and numerotation of the antigen receptors, creating a new science, immunoinformatics, at the interface between immunogenetics and bioinformatics [2]. IMGT has described a unique numbering system to universally define framework regions (FR) and CDR of IG and TR based on their conserved structure as follows: Cysteine 23 (1st-CYS) in FR1-IMGT, Tryptophan 41 (CONSERVED-TRP) in FR2-IMGT, hydrophobic amino acid 89, Cysteine 104 (2nd-CYS) in FR3-IMGT, Tryptophan/Phenylalanine 118 (J-TRP/J-PHE 118) in FR4-IMGT. Compared to the CDR3-IMGT, the JUNCTION includes the two anchors 104 and 118 [8, 9].

Studies in non-model organisms aiming to annotate antigen receptor gene loci and to characterize the expressed repertoire are often hampered by the lack of high-quality genome assemblies. In 2017, the dog became the third mammalian species for which all antigen receptor loci have been annotated [10]. The characterization of germline genes and expressed repertoire of T cell receptor loci in cats was first reported by Moore et al. in 2005 using cloning and Sanger sequencing of 31 TRG transcripts to identify 3 TRGV gene subgroups and 6 TRGJ gene variants [11]. Thus far, 8 feline TRG V genes assigned to 5 subgroups, 9 J genes and 6 C genes have been identified by mining of the NCBI TRACE Archive and Sanger sequencing of an expressed library [12, 13]. Another study analyzed the V gene germline repertoires of 48 mammalian species including the cat using the VgeneExtractor software on whole genome shotgun data [14]. Variable genes from 7 antigen receptor loci were catalogued and revealed that the cat has at least 46 TRAV, 20 TRBV, 5 TRGV and 7 TRDV genes. Next generation sequencing has been used in cats to characterize the expressed immunoglobulin repertoire [15]. However, neither the locus structure nor the expressed repertoire of the feline TR loci have been reported.

The cat is important both as a pet and as a large animal model for spontaneous diseases. The cat has been used as a naturally occurring animal model to study host-pathogen interactions in virus induced cancer caused by feline leukemia virus (FeLV) [16], as well as in an immunodeficiency syndrome caused by feline immunodeficiency virus (FIV) that resembles human immunodeficiency virus (HIV) [17]. The recent release of a high-quality genome assembly provides a basis for the annotation of antigen receptor gene loci in cats. Annotation would contribute to feline health as well as benefit the use of cats as a model for spontaneous diseases in humans [18].

The objectives of this study were to characterize the genomic organization and expressed repertoire of the feline TRA/TRD, TRB and TRG loci. We employed a Hidden Markov Model [19] approach to identify the feline TR germline genes and utilized high-throughput sequencing to characterize the feline expressed TR repertoire in lymphoid organs of normal cats. These findings will provide baseline data for the investigation of immune repertoires in pathologic conditions. Furthermore, the data will facilitate the development of improved molecular diagnostic tests for lymphoproliferative disorders, which are common diseases in domestic cats [11].

## Results

### TRG locus

**Fig. 1:**
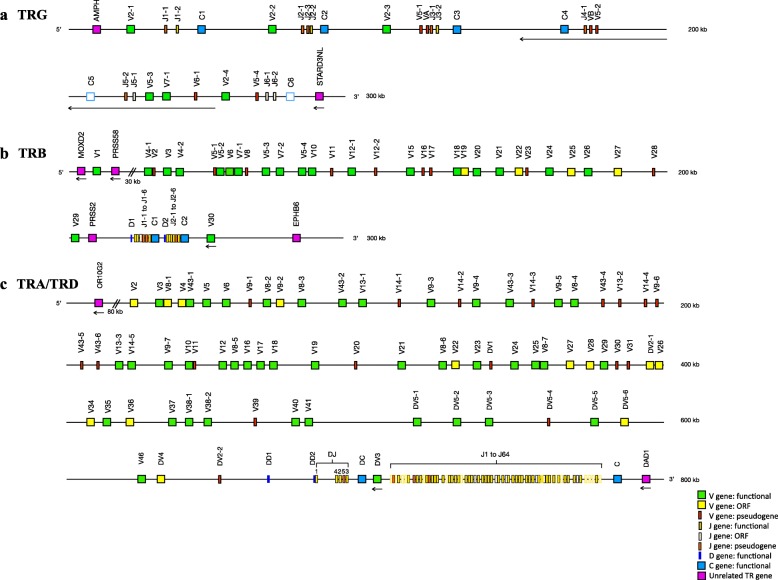
The genomic organization of feline T cell receptor loci; TRG (**a**), TRB (**b**) and TRA/TRD (**c**) deduced from the genome assembly Felis_catus_9.0. The diagram shows the position and nomenclature of all TR genes according to IMGT nomenclature. Boxes representing the genes are not to scale and exons are not shown. The arrows indicate an inverse transcriptional orientation. Magnifications of the TRB and TRA loci are provided in Additional file 1

**Fig. 2:**
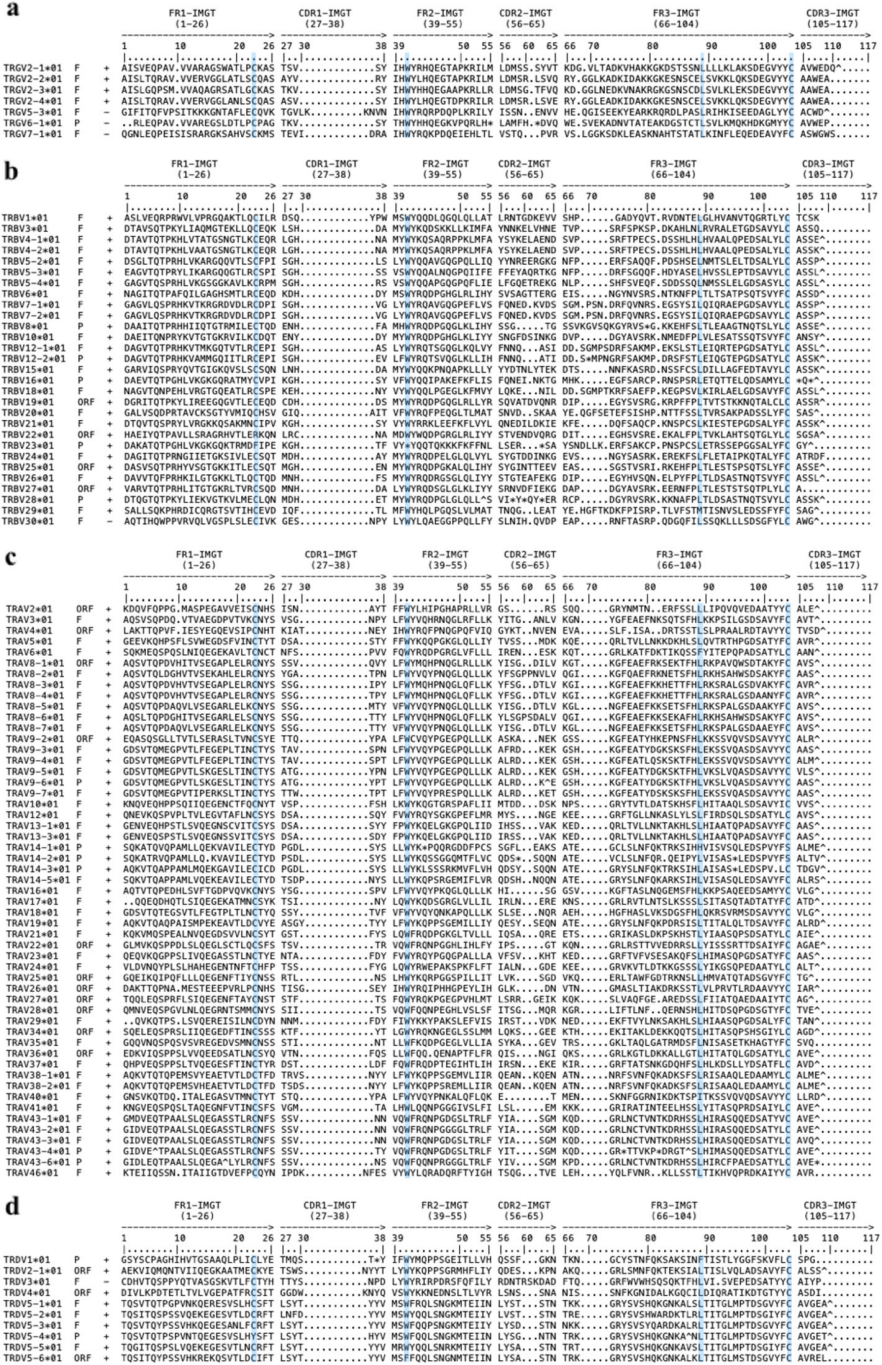
Alignment of deduced amino acid sequences of feline TRGV (**a**), TRBV (**b**), TRAV (**c**) and TRDV (**d**) genes. Only functional genes, ORF and in-frame pseudogenes are shown. Functionality and transcriptional orientation of the genes are indicated by ‘+’ and ‘-‘. The outline of complementarity determining regions (CDR-IMGT) and framework regions (FR-IMGT) are according to the IMGT unique numbering system for V-REGIONs. The four conserved amino acids are shaded in blue (1st-CYS23, CONSERVED-TRP41, hydrophobic AA 89 and 2nd-CYS104)

The *Felis catus* TRG locus spans approximately 260 Kb in the pericentromeric region of chromosome A2. The 5′ IMGT borne is Amphiphysin (AMPH, NCBI: XP_023105977.1) and the 3′ IMGT borne is a STARD3 N-Terminal Like gene homolog (STARD3NL, NCBI: XP_006929164.1) in an inverse transcriptional orientation (Fig. 1a). The TRG locus contains 30 genes: 12 TRGV (6 functional (F), 6 pseudogenes (P)), 12 TRGJ (4 F, 2 ORF (for open reading frame, IMGT functionality), 6 P), and 6 TRGC genes (4 F, 2 P) that are arranged in 5 complete and 1 incomplete V-J-(J)-C units (cassettes) (Table 1). The feline TRGV genes belong to 6 subgroups, two of them having 4 members (TRGV2 with 4 F, TRGV5 with 1 F and 3P) and the four other with a single member each; subgroup TRGV7 being the only one with a functional gene, subgroup TRGV6 containing two STOP-CODON in the V-REGION, TRGVA and TRGVB that are degenerate pseudogenes. The nucleotide identity between the different TRGV subgroups is 37.2–46.6%. The six functional TRGV genes are functional genes containing the conserved amino acid motif IHWY at the beginning of FR2-IMGT (positions 39–42) (Fig. 2a) [8, 20]. The canine TRGV1 and TRGV3 subgroup orthologs are absent in the cat [21]. The 12 feline TRGJ genes were designated based on the cassette they belong to. There are 4 functional TRGJ genes, 2 ORF and 6 pseudogenes. Each TRGC region is encoded by 3 exons (EX1, EX2A or EX2B and EX3) and all are functional except TRGC5 and TRGC6 due to frameshifts in EX1 and EX3 respectively.

**Table 1:** Number of feline TR genes in each locus and gene functionality

Gene	Functionality	Locus			
		TRA	TRB	TRG	TRD
V	F	37	20	6	5
	ORF	10	4		3
	P	15	9	6	3
D	F		2		2
	ORF				
	P				
J	F	41	8	4	2
	ORF	20	1	2	2
	P	3	3	6	1
C	F	1	2	4	1
	ORF				
	P			2	
Total		127	49	30	19

*F* functional gene, *ORF* open reading frame, *P* pseudogene

**Fig. 3:**
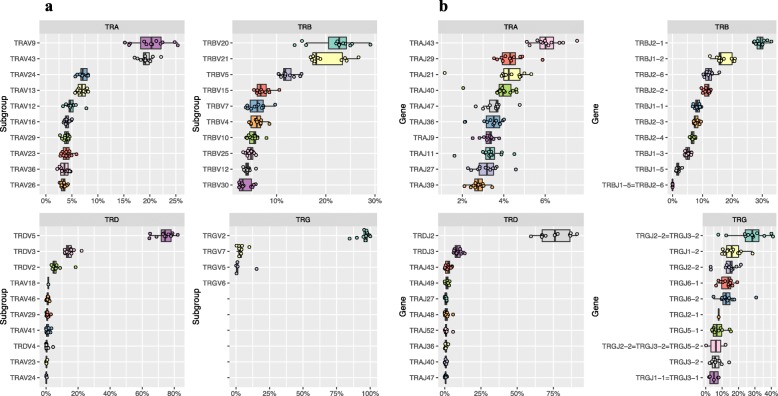
Box graphs showing V (**a**) and J (**b**) gene usage in each locus. X-axis shows the percentage of gene expression in a particular locus. Y-axis shows subgroup name (**a**) and gene name (**b**). Median, upper and lower quartile and outliers are indicated

**Fig. 4:**
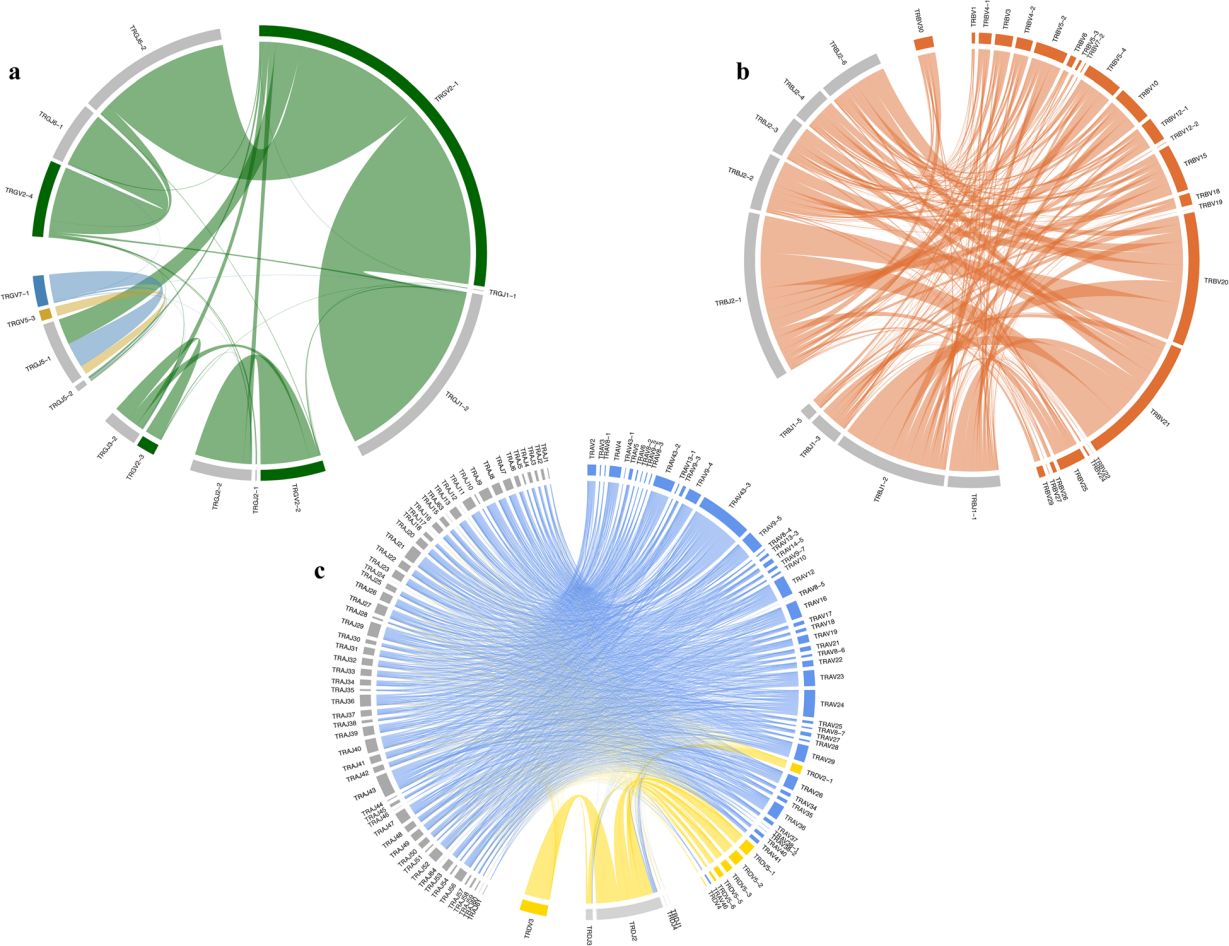
Circos plots showing V and J gene usage and pairing for the feline TRG (**a**), TRB (**b**) and TRA/TRD (**c**) loci. TRGV genes are colored by subgroup (**a**), TRBV genes are colored in orange (**b**), TRA/TRADV genes are colored by locus and J genes in all loci are colored in grey (**c**). The width of a link corresponds to the rearrangement frequency of a given V/J pairing. Genes are ordered according to their location on the chromosome

The overwhelming majority of all rearrangements in this dataset involved the four TRGV2 subgroup genes (median 97.1%) followed by TRGV7–1 and TRGV5–3 at 2.8 and 1.1%, respectively (Fig. 3a). No rearrangements involving the TRGV pseudogenes were found. For J genes, considerably less usage bias was seen (Fig. 3b). Distinction of rearrangements utilizing TRGVJ2–2 versus TRGVJ3–2 genes was frequently not possible because the two genes only differ by a single nucleotide at the 5′ end that is deleted in the majority of rearrangements (Fig. 3b). The TRGV and TRGJ genes in the same cassette preferentially rearrange versus those in a different cassette (Fig. 4a).

### TRB locus

The *Felis catus TRB* locus spans approximately 300 Kb on chromosome A2 and contains 33 TRBV (20 F, 4 ORF, 9 P), 2 TRBD (F), 12 TRBJ (8 F, 1 ORF, 3 P) and 2 TRBC (F) genes (Table 1). The 5′ and 3′ IMGT bornes are monooxygenase DBH-like 2 (MOXD2, NCBI ID: XM_003983120.4) in an inverted transcriptional orientation and EPH receptor B6 (EPHB6, NCBI ID: XM_023250648.1), respectively. The protease serine 58 gene (PRSS58, NCBI ID: XM_003983121.4) is located between the two most 5′ genes TRBV1 and TRBV4–1. The anionic trypsinogen gene (PRSS2, NCBI ID: XM_003983123.3) is located at the 3′ end of the locus, downstream of TRBV29 and upstream of the two D-J-C-clusters. The TRBV30 gene is located downstream of TRBC2 and in an inverted transcriptional orientation. Each D-J-C-cluster contains a single TRBD gene, six TRBJ genes and one TRBC gene (Fig. 1b and Additional file 1a).

The feline TRBV genes comprise 20 functional genes belonging to 17 subgroups (in a total of 27 subgroups), 4 ORF and 9 pseudogenes (Table 1). One TRBV subgroup (TRBV5) contains 4 members (3 F and 1 P), three TRBV subgroups (TRBV4 (2 F), TRBV7 (2 F) and TRBV12 (1F, 1P)) contain 2 members and the remaining subgroups contain one member. All feline TRBV genes were named based on their homology with canine orthologs except for the feline TRBV23 gene, which was named after the human ortholog due to the lack of a canine ortholog [22]. The four IMGT conserved amino acids, C23, W41, hydrophobic 89 and C104, were present in all functional TRBV genes (Fig. 2b). The feline TRBJ genes fall into 2 sets with 6 members each. Nine TRBJ genes are functional (8) or ORF (1) and contain the canonical FGXG amino acid motif (positions 118–121 in V-DOMAIN), and three are pseudogenes owing to containing a frameshift or STOP-CODON in the J-REGION. Two TRBD genes were named corresponding to their cluster and share 67.07% nucleotide identity. Similar to the situation in other mammals, the feline TRBC1 and TRBC2 genes have a high percentage of identity (98.0%), comprise 4 exons, and are both functional [22, 23].

High-throughput sequencing of the expressed repertoire revealed that the V genes TRBV20 (median 22.7%) and TRBV21 (18.1%) were preferentially utilized (Fig. 3a). TRBJ genes of the second D-J-C-cluster were more commonly rearranged than genes of the first cluster (cumulative medians of unambiguously called J genes 66.6% vs. 31.5%, respectively). In particular, TRBJ2–1 was utilized in 29.3% of all TRBJ rearrangements followed by TRBJ1–2 (16.0%), TRBJ2–6 (12.1%) and TRBJ2–2 (11.9%) (Fig. 3b).

### TRA/TRD locus

The *Felis catus* TRA and TRD loci are co-localized on a segment of approximately 800 Kb on chromosome B3 and consist of 62 TRAV (37 F, 10 ORF, 15 P), 64 TRAJ (41 F, 20 ORF, 3P), 1 TRAC (F), 11 TRDV (5 F, 3 ORF, 3 P), 2 TRDD (F), 5 TRDJ (2F, 2 ORF, 1P) and 1 TRDC (F) genes (Table 1). Several olfactory receptor (OR) genes (the nearest one, OR10G2, NCIB: XM_023255575.1) are located at the 5′ end of the feline TRA/TRD locus and the defender against cell death 1 (DAD1, NCBI: XM_019832791.2) gene is located at the 3′ end (IMGT 3′ borne) in inverted transcriptional orientation. Sequential TRA/TRD V genes are followed by a TRD D-J-C-cluster that is then followed by the most 3′ TRDV3 gene in an inverted transcriptional orientation. Downstream of this block is the cluster of TRAJ genes followed by a single TRAC gene (Fig. 1c and Additional file 1b).

The 62 feline TRAV genes belong to 38 subgroups, 32 subgroups containing a single gene (19 subgroups with one F gene, 8 with one ORF and 5 with one P) and 6 subgroups containing multiple genes (for a total of 18 F, 2 ORF and 10 P). The feline TRAV2, TRAV3, TRAV4 and TRAV5 were named after the human orthologs due to the lack of a canine ortholog. The 11 feline TRDV genes belong to 5 subgroups and comprise 5 functional genes, 3 ORF and 3 pseudogenes (Table 1). The four conserved IMGT amino acids of the V-REGION, C23, W41, hydrophobic 89 and C104, are present in all functional feline TRAV and TRDV genes (Fig. 2c-d). Of the five TRDJ genes, two are F and two are ORF and contain the canonical FGXG motif (positions 118–121 in V-DOMAIN); the last one is a pseudogene. Of the 64 TRAJ genes, 41 are functional, 20 are ORF and 3 are pseudogenes. The genes TRAJ29 and TRAJ51 were named based on the human orthologs because no canine orthologs exist. Orthologs for the feline genes TRAJ62, TRAJ63, TRAJ64 and TRAJ65 do not exist in dogs nor in humans. The two feline TRDD genes are functional and share only 58.0% identity. The TRDC and TRAC genes are functional and comprise 4 exons.

The TRA V and J gene usage was relatively balanced compared to other feline TR loci. The most commonly expressed V gene subgroups were TRAV9 and TRAV43, utilized in 20.3 and 19.1% of rearrangements, respectively (Fig. 3a). All other functional genes were rearranged at a frequency less than 7%. In contrast, gene usage was more biased in the feline TRD locus. The most frequently rearranged V genes were the TRDV5 subgroup genes (median 74.3%) followed by the TRDV3 gene (13.7%). The expressed repertoire was dominated by TRDJ2 (76.1%) which is an ORF gene. The TRA/TRD V genes almost exclusively rearranged to J genes within their locus (Fig. 4c).

### Recombination signal (RS) sequences

**Fig. 5:**
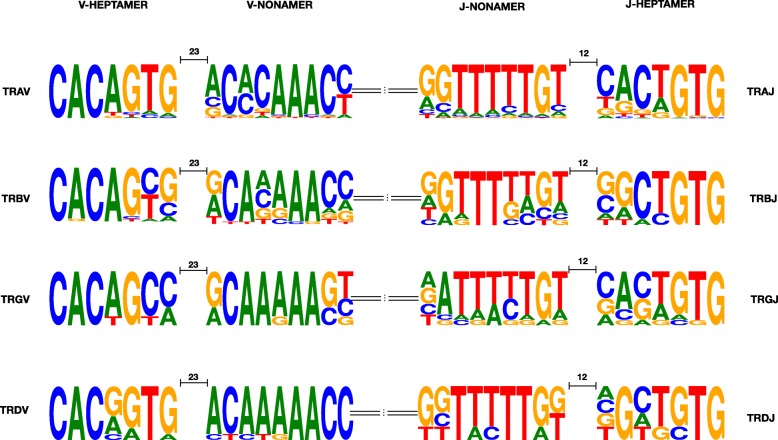
Position weight matrixes of recombination signal sequences of functional T cell receptor V and J genes. The height of symbols indicates the relative frequency of each nucleotide at that position

The first three nucleotides (cac) of the heptamer and the poly-A tract of the nonamer of functional V and J genes were highly conserved in all feline TR loci. The following two positions of the heptamer and an individual position of the nonamer were less conserved. Thymine and guanine at the last two nucleotides of the V-HEPTAMER were conserved in the feline TRA and TRD loci. The seventh nucleotide of the J-HEPTAMER of feline TRG was highly diverse. The cytosine at the sixth residue of the J-HEPTAMER was notably conserved in the feline TRD locus (Fig. 5).

### Phylogenetic analysis of the V-REGION

**Fig. 6:**
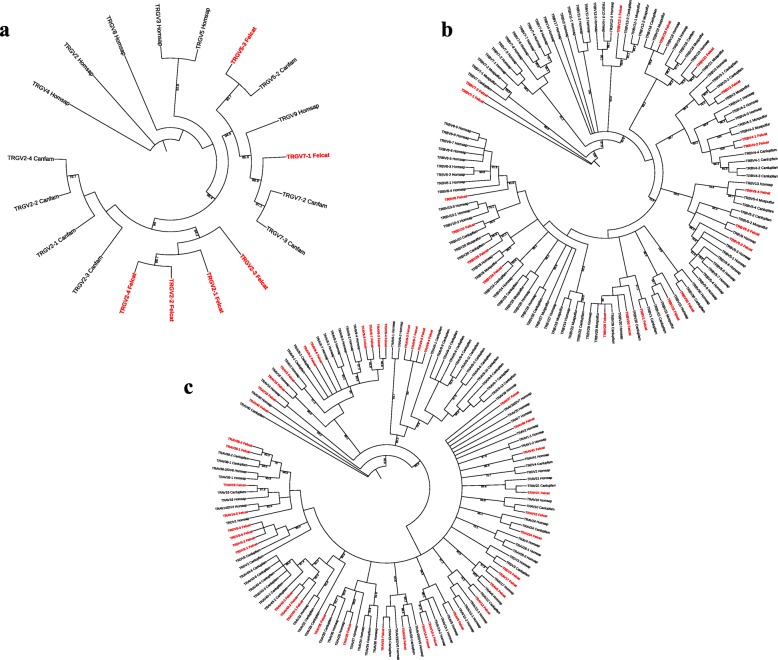
Phylogenetic analysis of functional TRG (**a**), TRB (**b**) and TRA/TRD (**c**) V genes. Unrooted trees were constructed using the neighbor-joining method based on V-REGION nucleotide sequences of dog, ferret (TRB) and human. The percentages of the nodes in 1000 bootstrap replicates are shown on the branches. Feline genes are labelled in red. The IMGT standardized abbreviation for taxon is used: six letters for species (Homsap, Felcat) and nine letters for subspecies (Canlupfam, Musputfur)

To investigate the evolutionary relationship of functional T cell receptor V genes, feline V-REGION sequences and ortholog genes were aligned, and unrooted trees of each TR locus were constructed using the neighbor-joining method. Feline T cell receptor V genes form monophyletic clades with their canine and ferret orthologs (Fig. 6). Clustering of multimember subgroups of different orthologs is frequently observed in V genes located close to the 5′ end of TR loci as seen with the TRGV2, TRBV7 and TRAV9 subgroups. Single member V gene subgroups forming monophyletic clades with their corresponding orthologs are commonly found throughout the TR loci.

## Discussion

The structure of the TRG locus differs considerably across species. Rabbits and humans have one TRG locus, with V genes being located upstream of one and two J-C-clusters, respectively, whereas ruminants possess two TRG loci with multiple cassettes distantly located on the same chromosome [24–26]. The feline TRG locus most closely resembles that of the dog, which has 8 V-J-(J)-C cassettes [27]. The fact that cassettes 4 and 5 are in an inverted orientation in the cat, despite a high homology to dog V genes, suggests that the inversion likely occurred after speciation. Interestingly, the vast majority of V genes used were of the TRGV2 family. The reason for the biased usage of the TRGV2 genes is unclear but could be due to the physical proximity of the V and J genes. Indeed, in humans, TRGV9 (the functional V gene most in 3′) is located closest to TRGJP (the functional J gene most in 5′) and is the most highly expressed in adult peripheral blood [26, 28–30]. However, whereas the genomic cassette structure favors physical V and J proximity, it also makes the expression strongly dependent on the functionality of the constant gene, and this may explain the poor expression of TRGV5–1 and TRGV7–1 which are associated with the pseudogene TRGC5 (Fig. 4a). Of note, TRGJx-2 genes were more frequently rearranged (more than 80%) than TRGJx-1 genes, where TRGJx-1 and TRGJx-2 refer to the first and second J gene in each TRG cassette, respectively. This is in line with the finding that 4 out of 5 TRGJx-2 genes are functional while 4 out of 6 TRGJx-1 genes are pseudogenes (Fig. 1a). In fact, almost none of the four TRGJx-1 pseudogenes (TRGJ1–1 to TRGJ4–1) were found to rearrange whereas the two ORF genes TRGJ5–1 and TRGJ6–1 did rearrange at a rate comparable to that of TRGJx-2 genes (Fig. 4a).

The feline TRB locus is structurally similar to that of humans, dogs, ferrets and rabbits loci in regard to the 5′ and 3′ borne genes and the presence of two D-J-C-clusters. In contrast, artiodactyl species possess 3 D-J-C-clusters [22, 23, 31–34] (Fig. 1b). Compared to the human TRB locus that contains 68 V genes with 48 functional genes, the feline TRB locus contains only 33 V genes including 9 pseudogenes [35]. The overall lower number of genes in the feline TRB locus is reflected by fewer multigene subgroups and fewer genes per multigene subgroup (Fig. 1b). The duplication of feline TRBV genes was more common near the 5′ end of the locus, which is similar to the canine, ferret and rabbit TRB loci [22, 23, 33]. TRBV gene showed preferential usage but less than observed for the TRGV genes. TRBJ genes of the second D-J-C-cluster were more commonly rearranged than genes of the first cluster. The preferential usage of particular V and J genes are well-documented features of the TRB repertoire in other vertebrates [36–38]. More specifically, expression analysis of human TRB genes showed preferential use of TRB J genes in the second over the first D-J-C-cluster, as also seen in the cat (Fig. 4b) [39].

Comparative genomic analysis demonstrates that the feline TRA/TRD loci share similar organization to human, mouse and canine TRA/TRD loci, with small differences in the numbers of V, D and J genes [10, 40]. Gene duplications were more frequent at the 5′ end of the locus, similar to the canine TRA locus [10]. The larger number of feline versus canine TRDV genes is due to duplications of the TRDV5 gene (TRDV5–1 to TRDV5–6). Interestingly, a TRDV gene that had previously not been identified in other mammals and that shared 52.1–55.1% nucleotide identity with TRDV2 was found between TRDV5–6 and TRDV4 (Fig. 1c). Owing to its localization, it was the first desinated as was TRDV6 and classified as ORF. However this gene is functional when assigned to TRAV46, single member of a new subgroup in feline TRA locus (since then, a pseudogene TRAV46 has been identified in rabbit, Rhesus monkey and dog) [27]. The TRA V and J gene usage was relatively balanced compared to other feline TR loci. Interestingly, gene of the TRAV43 subgroup, which was only been identified in the dog and the cat, were the second most frequently expressed TRA gene. Gene usage was more biased in the feline TRD locus. The feline TRDV5–1 gene was previously classified as a pseudogene due to an alternative INIT-CODON (ctg instead of atg) but it was the second most utilized V gene in the TRD transcript repertoire and currently assigned as functional gene (Fig. 4c). It is unclear how this affects protein translation and expression. The TRA/TRD V genes almost exclusively rearranged to J genes within their locus. In humans, the TRDV1, TRDV2 and TRDV3 genes are almost exclusively used in TRD rearrangements while TRAV14/DV4, TRAV29/DV5 and TRAV23/DV6 genes can rearrange to either TRA or TRD J genes [41, 42]. The human TRD repertoire becomes more limited with age and preferential expression of particular genes is apparent in different organs [43]. In this study, samples came from young cats only, which precludes assessment of age-associated repertoire features.

The first three nucleotides of the heptamer and the poly-A tract of the nonamer were highly conserved in all feline TR loci and were consistent with the canonical RS described for other species [6, 44]. Thymine and guanine at the last two nucleotides of the V-HEPTAMER were conserved in the feline TRA and TRD loci similar to humans and mice. These conserved nucleotides reflect the close relationship of V genes in the TRA/TRD loci and are possibly critical for the rearrangement process of functional TRAV and TRDV genes. However, the seventh nucleotide of the J-HEPTAMER of feline TRG was highly diverse compared to the respective position in humans and mice, which have a conserved thymine [45]. The first three nucleotides of the heptamer play a crucial role in the recombination process and divergence of these positions can impair or preclude gene recombination [46]. Consequently, the IMGT functionality criteria require a canonical RS at the 3′ end of V genes (V-HEPTAMER) and the 5′ end of J genes (J-HEPTAMER) for genes to be classified as functional genes [47]. To what extent ORF gene, i.e. genes with non-canonical V-HEPTAMER or J-HEPTAMER sequence, rearrange is unknown but likely depends on the degree of divergence.

Overall, phylogenetic analyses showed that human, dog and cat T cell receptor V genes extensively intermingle rather than forming separate clades, and the multigene family clustering suggests evolutionary dynamics shared among these species. It is thought that multigene families such as major histocompatibility genes, immunoglobulin genes and T cell receptor genes are subject to birth-and-death evolution, which explains the emergence of new genes by repeated gene duplication and that some genes are retained in the genome for a long time, while others are deleted or become pseudogenes by extensive mutations [48, 49]. This model may fit the TRB and TRA loci that showed clustering of orthologs in multimember subgroups. For example, 1 out of 4 V genes in the feline TRBV5 subgroup is a pseudogene, 2 out of 4 V genes in the canine and ferret TRBV5 subgroup are pseudogenes and 1 out of 8 V genes in the human TRBV5 subgroup is a pseudogene [50]. Of note, the feline TRAV43 subgroup is not found in humans and Rhesus monkeys, which might indicate that the gene has evolved after the divergence of primates. Some of the feline V genes such as TRAV37 and TRAV46 are outgroups, possibly suggesting that the genes recently evolved as functional genes in domestic cats. Gene duplication of ancestral genes, followed by diversification through mutation, is possibly the major mode of evolution of T cell receptor V genes. This evolutionary mechanism likely contributes to locus complexity and promotes diversity of immune cells. This evolution model of TR genes has also been observed in other species [23, 36, 51].

## Conclusion

Our study describes, for the first time, the genomic organization of all the feline TR loci, and characterizes the expressed repertoire in normal lymphoid organs using high-throughput sequencing. The genomic organization of the feline TR loci was generally similar to that of previously described TR loci [40, 52]. The feline TRG locus showed the most inter-species variation, but closely resembles the TRG locus in dogs with multiple V-J-(J)-C cassettes. The analysis of expressed antigen receptor sequences revealed a biased V/J usage, mainly with the feline TRG and TRD genes. The relatively high number of functional TRDV genes and preferential gene rearrangements in TRG and TRD loci may contribute to repertoire diversity and bias of γδ T cells, consistent with their genomic structure and more innate-like function, sensing early events of cellular stress and infection [48]. While factors such as age, infection, immune disorders and cancer impact the diversity of the TR repertoire [49, 51, 53], knowledge of the TR repertoire in normal cats is fundamental to our understanding of adaptive immunity in this species. This should benefit future research on disease pathogenesis, clinical diagnostics and immune therapy in feline medicine.

## Methods

### Germline gene identification

Germline genes were identified using the flexible Hidden Markov Model StochHMM [19] as well as a blast-based approach [54]. The Hidden Markov Model StochHMM approach has been described elsewhere [55]. Briefly, model states corresponded to the nucleotide positions of the IMGT unique numbering system [8]. Emission and transition probabilities were generated based on antigen receptor gene sequence data of various species obtained from the IMGT/GENE-DB database [45]. The feline TR loci were then searched in the Felis_catus_9.0 reference genome (GenBank assembly accession: GCA_000181335.4). To complement the HMM based approach, canine and human TR genes were blasted against the putative feline locus sequences and hits were assessed manually. Boundary (5′ and 3′ IMGT bornes) and intercalated genes not related to TR loci were identified by comparison of feline TR loci with annotated ortholog species on ENSEMBL database (https://uswest.ensembl.org).

### Nomenclature and gene functionality

Gene names and subgroups as well as gene functionality were assigned based on IMGT nomenclature rules, and in collaboration with IMGT [47, 56]. Gene names and subgroup numbers were assigned primarily according to nucleotide percent of identity to canine orthologs and more than 75% nucleotide identity cut-offs. Human ortholog assignments were used when no canine ortholog was found, and new gene nomenclature was assigned if no orthologs were identified in other documented species. The IMGT nomenclature system was also applied to all gene names, based on nucleotide percent of identity and relative localization in the locus [2]. Gene functionality was predicted in accordance with IMGT criteria [47]. IMGT gene names approvals from this work are reported in IMGT-NC reports #2019–2-0111 (25 TRG), #2019–4-0116 (46 TRB) and #2019–6-0218 (142 TRA/TRD). Additional TRG genes are reported in IMGT-NC report #2019–3-0111 by Burnett, Avery and Rout [57].

### Sequence alignments of TR V-REGION and RS

Deduced amino acid sequences of predicted functional genes, ORF and in-frame pseudogenes were manually aligned according to the IMGT unique numbering of V-REGION [8]. Deduced amino acid alignments of functional genes, ORF and gapped pseudogenes were generated using StochHMM. Recombination signal (RS) sequences of potentially functional V and J genes of each TR locus were aligned using Geneious (9.1.7) software [58] and depicted as sequence logos by WebLogo [59].

### Animals and RNA extraction

Three specific-pathogen-free 3-month-old domestic cats were obtained from the breeding colony of the Feline Research Laboratory (FRL) in the Feline Nutritional Center, University of California Davis (UC Davis). Cats were cared for as regulated by the Institutional Animal Care and Use Committee (IACUC) of the University of California, Davis USA (Animal Welfare Assurance Number A3433–01). They were housed in open rooms in the facilities of the FRL, fed a dry maintenance diet with ad libitum access to fresh water, and provided with environmental enrichment (toys, scratching posts, daily petting and weekly brushing). The facility maintains room temperatures between 18 and 24 **°**C, and has a 14 h light/10 h dark cycle. The cats were euthanized with intravenous sodium pentobarbital overdose, in accordance with the IACUC protocol and the American Veterinary Medical Association (AVMA) Guidelines for Euthanasia of Animals. Thymus, spleen and mesenteric lymph node were harvested and stored at -80C. Total RNA was extracted using the RNeasy Mini kit, according to the manufacturer’s recommendations (Qiagen, MD, USA). Biological and technical replicates were done on 3 target organs from one of the cats. The RNA integrity number (RIN) was assessed by the UC Davis Genome Center using the 2100 Bioanalyzer (Agilent Technology, CA). Each RNA sample was assessed in order to ensure the RIN was higher than 8.

### T cell receptor library preparation for HTS

Primer sequences and concentration used in the experiments are shown in Additional file 2. Reverse primers targeting the 5′ end of the constant region were designed for each TR locus. Multiple sequence alignments were done for loci with multiple C genes, and consensus primers were chosen whenever possible. An overhang sequence was attached to the 5′ end of each primer, as described in the manufacturer’s protocol for Illumina library preparation running on a MiSeq platform with V3 reagents [60]. The rapid amplification of cDNA 5′ ends (5’RACE) technique was performed to obtain the full-length junctional sequence [61]. First strand cDNA synthesis was primed using oligo-dT primers and anchored switching oligonucleotide ligation at the 5′ end of the mRNA template. Approximately 1 μg of total RNA was converted to cDNA using SMARTScribe reverse transcriptase (Takara, CA) following the manufacturer’s protocol. Amplification was carried out for each locus separately using the previously described reverse primers and a universal forward primer. The KAPA HiFi HotStart ReadyMix PCR kit (KAPA Biosystems, MA) was used in a 50 uL reaction volume and cycling conditions as follows: 95 °C for 3 min; 25 cycles of 20 s at 98 °C, 15 s at 64 °C, and 30 s at 72 °C; 72 °C for 3 min. Amplified products were pooled followed by purification and size selection using SPRIselect beads (Beckman Coulter Inc., CA). Dual barcode adapters were attached using a second round of PCR as outlined in the Illumina library preparation protocol [60]. The libraries were quantified, pooled at equimolar ratios and submitted to the DNA Technology Core, UC Davis (Davis, CA) for paired-end 2 × 300 bp sequencing using the Illumina MiSeq system (Illumina, Inc., CA). The sequence data is available through the Sequence Read Archive submission number SUB5687466.

### Data analysis

Raw fastq files were quality trimmed with Trimmomatic software using a sliding window algorithm and a Q30 cut-off [62]. Paired read joining, identification of V, D and J genes and extraction of junctional regions were done using the Interrogate/ARResT platform based on the feline germline genes identified in this study [63]. Statistical analysis was done using R software and V and J pairing was visualized by Circos software [64].

### Phylogenetic analyses

Functional human, ferret and dog V-REGION nucleotide sequences were retrieved from the IMGT/LIGM-DB (http://www.imgt.org) database (nucleotide sequence accession numbers are shown in Additional file 3). Feline V-REGION nucleotide sequences were obtained via this study. The sequences corresponding to functional IMGT V-REGION were aligned and phylograms were constructed using a Neighbor-joining method with 1000 bootstrap replicates and the Geneious software (9.1.7). The outputs were visualized using the Interactive Tree of Life software [65].

## Supplementary information

**Additional file 1.** Magnification of the genomic organization of feline T cell receptor loci; TRB (a), and TRA/TRD (b).

**Additional file 2.** Table showing primer sequences and concentration used for TR library preparation.

**Additional file 3.** Reference sequences used for phylogenetic analyses.

## Data Availability

The Felis_catus_9.0 reference genome was obtained from the National Center for Biotechnology Information Assembly database using the accession number GCA_000181335.4. IMGT/V-QUEST and IMGT/HighV-QUEST IMGT reference directory releases for *Felis catus* TR are the following: *Felis catus* TRG [Release 201908–4 (21 February 2019)], *Felis catus* TRB [Release 201910–2 (5 March 2019)], *Felis catus* TRA and TRD [(Release 201927–2 (2 July 2019)]. IMGT reference directory sets are provided per group and per taxon at http://www.imgt.org/vquest/refseqh.html#VQUEST or can be downloaded at http://www.imgt.org/about/downloads.php. Sequences of the *Felis catus* loci annotated in IMGT/LIGM-DB: IMGT000045 (828158 bp) for the TRA/TRD locus from chromosome B3, IMGT000037 (302423 bp) for the TRB locus from chromosome A2 and IMGT000036 (296001 bp) for the TRG locus from chromosome A2 are contiguous without gaps. The reference sequences used for phylogenetic analyses were downloaded from NCBI and IMGT/LIGM-DB using the accession numbers listed in Additional file 3.

## Abbreviations

C: Constant gene
CDR: Complementarity determining region
Cys: Cysteine
D: Diversity gene
EX: Exon
F: Functional gene
FR: Framework region
HTS: High-throughput sequencing
IG: Immunoglobulin
IMGT: International ImMunoGeneTics
J: Joining gene
ORF: Open reading frame
P: Pseudogene
RS: Recombination signal
TR: T cell receptor
TRA: T cell receptor alpha
TRB: T cell receptor beta
TRD: T cell receptor delta
TRG: T cell receptor gamma
V: Variable gene
W: Tryptophan
