# Supplementary figures and images for: Topology and expressed repertoire of the Felis catus T cell receptor loci
Source: BMC Genomics. 2020 Jan 6;21:20. doi: 10.1186/s12864-019-6431-5 (PMC6945721; doi:10.1186/s12864-019-6431-5)

**a TRBD, TRBJ and TRBC genes**

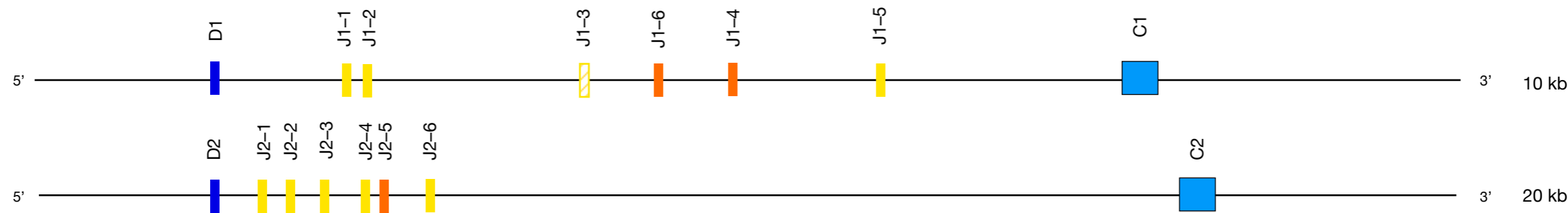

**b TRAJ genes**

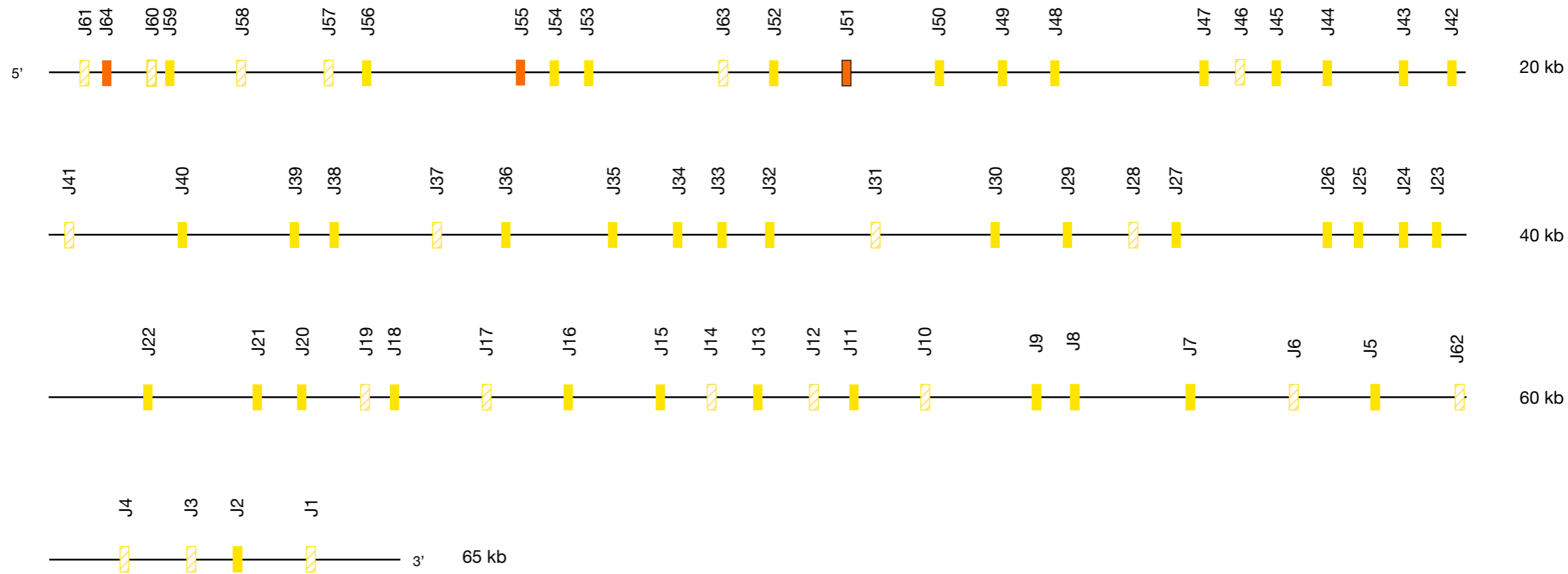

Supplement: Supplementary file 1 — Additional file 1. Magnification of the genomic organization of feline T cell receptor loci; TRB (a), and TRA/TRD (b). [file 12864_2019_6431_MOESM1_ESM.pdf]
