# Supplementary material for: Topology and expressed repertoire of the Felis catus T cell receptor loci
Source: BMC Genomics. 2020 Jan 6;21:20. doi: 10.1186/s12864-019-6431-5 (PMC6945721; doi:10.1186/s12864-019-6431-5)
Supplement: Supplementary file 2 — Additional file 2. Table showing primer sequences and concentration used for TR library preparation. [file 12864_2019_6431_MOESM2_ESM.docx]

| Primer | **Sequence ^a^**  **(5’ 🡪 3’)** | **Final concentration**  **(pmol/ul)** | |
| --- | --- | --- | --- |
| First strand cDNA synthesis | | |  |
| Oligo_dT27 | TTTTTTTTTTTTTTTTTTTTTTTTTVN | 0.6 | |
| Switching oligo ^b^ | AAGCAGTGGTATCAACGCAGAGTACTCTT(rG)_5_ | 1.2 | |
| Specific gene amplification | | |  |
| TSO_F55_OH | **TCGTCGGCAGCGTCAGATGTGTATAAGAGACAG**AGCAGTGGTATCAACGCAGAGT | 0.5 | |
| feTRAC_7R59_OH | **GTCTCGTGGGCTCGGAGATGTGTATAAGAGACAG**TGGTACACGGCAGGGTCAGGGTTCT | 0.12 | |
| feTRBC_13R55_OH | **GTCTCGTGGGCTCGGAGATGTGTATAAGAGACAG**TGACCTTGGGAGGGCTCACCT | 0.12 | |
| feTRDC_4R54_ OH | **GTCTCGTGGGCTCGGAGATGTGTATAAGAGACAG**TGGTTTGGCAGGAGGCTGGC | 0.12 | |
| feTRGC1_26R58_OH | **GTCTCGTGGGCTCGGAGATGTGTATAAGAGACAG**GGAAGAAAAATCGTGGGCTTGGGG | 0.032 | |
| feTRGC2_25R59_OH | **GTCTCGTGGGCTCGGAGATGTGTATAAGAGACAG**GGAAGAAAAATTGTAGGCTTTGGGG | 0.032 | |
| feTRGC3_26R58_OH | **GTCTCGTGGGCTCGGAGATGTGTATAAGAGACAG**GGAAGAAAAATTGTGGGCCTGGGG | 0.032 | |
| feTRGC4_26R58_OH | **GTCTCGTGGGCTCGGAGATGTGTATAAGAGACAG**GGAAGAAAGAGTGTGGGCTTGGGG | 0.032 | |
| Barcode and sequencing adapter attachment ^c^ | | |  |
| Adapter read 1 | CAAGCAGAAGACGGCATACGAGATXXXXXXXXGTCTCGTGGGCTCGG | 0.3 | |
| Adapter read 2 | AATGATACGGCGACCACCGAGATCTACACXXXXXXXXTCGTCGGCAGCGTC | 0.3 | |

**Table showing primer sequences and concentration used for TR library preparation**

^a^ Overhang sequences are indicated in bold

^b^ Primer was modified from [61].

^c^  xxxxxxxx denotes optional sequence of sample barcode

[61] Mamedov IZ, Britanova OV, Zvyagin IV, Turchaninova MA, Bolotin DA, Putintseva EV, Lebedev YB, Chudakov DM: Preparing unbiased T-cell receptor and antibody cDNA libraries for the deep next generation sequencing profiling. Front Immunol 2013, 4:456.
