# Supplementary material for: Topology and expressed repertoire of the Felis catus T cell receptor loci
Source: BMC Genomics. 2020 Jan 6;21:20. doi: 10.1186/s12864-019-6431-5 (PMC6945721; doi:10.1186/s12864-019-6431-5)
Supplement: Supplementary file 3 — Additional file 3. Reference sequences used for phylogenetic analyses. [file 12864_2019_6431_MOESM3_ESM.docx]

Reference sequences used for phylogenetic analyses

- **Human TRG locus** (NCBI ID): AF159056
- **Human TRB locus** (NCBI ID): NG001333
- **Human TRA/TRD locus** (NCBI ID): AE000521
- **Canine TRG locus** (IMGT ID): IMGT000006
- **Canine TRB locus** (IMGT ID): IMGT000005
- **Canine TRA/D locus** (IMGT ID): IMGT000004
- **Ferret TRB locus contig** (IMGT ID): IMGT000022, IMGT000023
